# Protective Effect of *Plantago major* Extract against *t*-BOOH-Induced Mitochondrial Oxidative Damage and Cytotoxicity

**DOI:** 10.3390/molecules201017747

**Published:** 2015-09-25

**Authors:** Joyce C. Mello, Mariano V. D. Gonzalez, Vivian W. R. Moraes, Tatiana Prieto, Otaciro R. Nascimento, Tiago Rodrigues

**Affiliations:** 1 Centro de Ciências Naturais e Humanas, Universidade Federal do ABC, Santo André, SP 09210-170, Brazil; E-Mails: joyce.cmello@gmail.com (J.C.M.); viviwerloger@yahoo.com.br (V.W.R.M.); tati_prieto@yahoo.com.br (T.P.); 2 Centro Interdisciplinar de Investigação Bioquímica, Universidade de Mogi das Cruzes, Mogi das Cruzes, SP 08780-911, Brazil; E-Mail: marianovalio@yahoo.com.br; 3 Grupo de Biofísica Molecular “Sergio Mascarenhas”, Departamento de Física e Ciência Interdisciplinar, Instituto de Física de São Carlos, Universidade de São Paulo (USP), São Carlos, SP 13566-590, Brazil; E-Mail: ciro@if.sc.usp.br

**Keywords:** antioxidant, free radicals, oxidative stress, plantain, folk medicine

## Abstract

*Plantago major* L. produces several chemical substances with anti-inflammatory and analgesic activities and its use in the treatment of oral and throat inflammation in popular medicine is well described. In this study, the antioxidant potential of the *Plantago major* hydroethanolic extract was screened and its protective action was evaluated against *t*-BOOH-induced oxidative stress. The extract was obtained by fractionated percolation using 50% ethanolic solution and, after drying, suspended in dimethyl sulfoxide. The chromatographic profile of crude extract was obtained with the identification of some phytochemical markers and the total phenols and flavonoids were quantified. The scavenger activity against DPPH (1,1-diphenyl-2-picrylhydrazyl) radicals was determined and the antioxidant activity in biological systems was evaluated in isolated rat liver mitochondria and HepG2 cells. The extract exhibited a significant free radical scavenger activity at 0.1 mg/mL, and decreased the ROS (reactive oxygen species) generation in succinate-energized mitochondria. Such an effect was associated with the preservation of the intrinsic antioxidant defenses (reduced glutathione and NAD(P)H) against the oxidation by *t*-BOOH, and also to the protection of membranes from lipid oxidation. The cytoprotective effect of PmHE against *t*-BOOH induced cell death was also shown. These findings contribute to the understanding of the health benefits attributed to *P. major*.

## 1. Introduction

Oxidative damage as a result of the action of free radicals is considered the major cause of aging [1] and the emergence of many diseases [2], including diabetes [3] and cancer [4]. A small fraction of oxygen consumed by the mitochondrial respiratory chain in oxidative phosphorylation is partially reduced to superoxide anion that in some conditions may be converted to hydrogen peroxide and hydroxyl radicals which are able to damage mitochondrial lipids, proteins and DNA [5,6]. Thus, mitochondria are an interesting biological model for studies involving free radicals and antioxidants since they are, simultaneously, source and target of free radicals [7,8].

Due to the large number of substances with antioxidant potential synthesized during secondary metabolism of plants, antioxidants derived from medicinal plants have been studied extensively in search of new compounds with the capacity to decrease the production or eliminate free radicals produced [9,10]. In this sense, the study of antioxidant activity in plants commonly used in folk medicine must be considered, since many effects attributed to plants may have antioxidant contribution of efficacy such as anti-inflammatory and hepatoprotective effects [11,12,13,14].

*Plantago major* L. (Plantaginaceae) is an herbaceous plant found naturally in regions of subtropical and temperate climate and it is easily cultivated in Brazil [15,16]. *P. major* extracts are often used in traditional medicine to treat oral wounds and throat inflammation, due to its anti-inflammatory, analgesic, and antipyretic effects [16,17,18].

Studies in polar extracts of other *Plantago* species have described the antioxidant capacity of this genus, which was attributed to the presence of compounds with free radical scavenger activity [19,20]. Considering that antioxidant activity can contribute to the anti-inflammatory properties associated with the popular use of this plant, in this work the antioxidant capacity of the hydroethanolic extract of *P. major* was evaluated, in order to verify whether the habitual use of alcoholic solutions to obtain plant extracts in popular Medicine are capable of providing antioxidant benefits.

## 2. Results and Discussion 

Different cellular responses against intrinsic or extrinsic stimuli may increase the production of reactive oxygen and nitrogen species and result in oxidative/nitrosative stress, a complex and multifactorial process that involves the formation of oxidized intermediates and final products [2,6]. The extension of the damage achieved during the oxidation process depends on the type of the free radicals and reactive species produced, since they have different reactivity, oxidation potential, solubility, and molecular targets [21]. Therefore, an intricate antioxidant defense system maintains the cellular redox homeostasis [21]. This system comprises antioxidant enzymes and small molecules, complemented by exogenous antioxidants obtained orally [22,23]. Polyphenols, including flavonoids, are abundant secondary metabolites produced by plants with antioxidant activity, and suitable extractive methods are needed to obtain such compounds in their active form [9,10,24,25].

In order to standardize the *P. major* extract, firstly the amount of total phenols and flavonoids contained in 0.1 mg/mL *P. major* hydroethanolic extract (PmHE) were quantified. PmHE presented 4.48% (*w*/*w*) of total phenols and 0.29% (*w*/*w*) of flavonoids. Although widely used in popular medicine, extraction using ethanolic solution has been poorly studied in the *Plantago* genus as methanol is ordinarily used. The amount of phenols in PmHE were lower than those observed in methanolic extracts of *P. major*, whose reported concentration was 9.17% [26], or methanolic extracts of other genus, such as *P. lagopus* that presented 7.99% [27].

**Figure 1:**
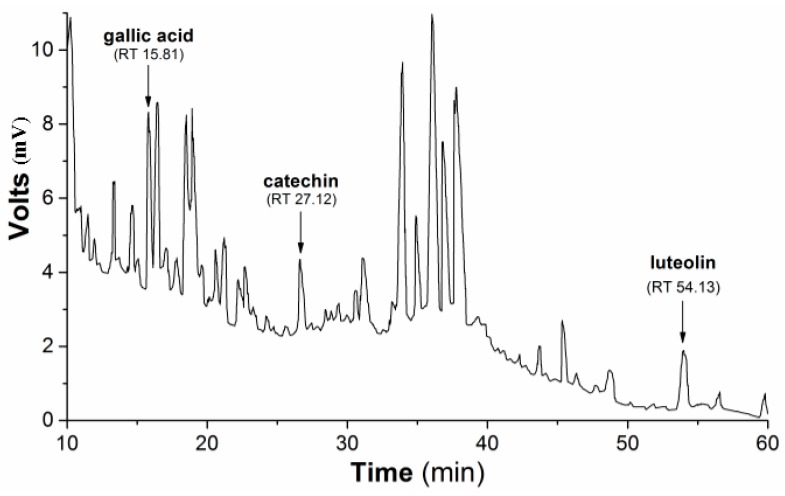
HPLC separation profile of *P. major* hydroethanolic extract. Representative chromatogram of the isolated compounds/fractions with detection at 245 nm. Peaks indicate the retention times of some flavonoids, which were identified by comparison with flavonoid standards.

Subsequently, the HPLC (high-performance liquid chromatography) separation profile of PmHE was obtained as described in the Methods Section using dry extract suspended in DMSO (dimethyl sulfoxide) and injected in the same proportion as used in the determination of antioxidant activity assays. The qualitative identification using standard solutions confirmed the presence of gallic acid, catechin, and luteolin (Figure 1). The flavonoid luteolin is a chemical marker in the *Plantago* genus [28,29].

**Figure 2:**
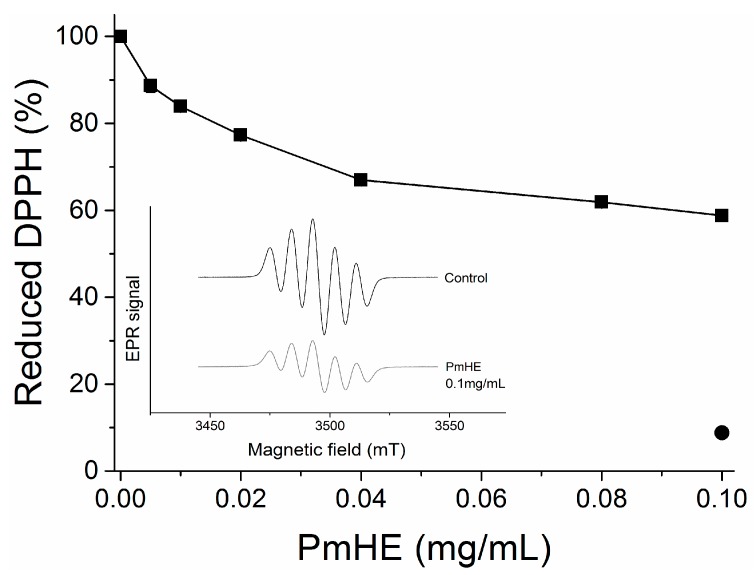
1,1-Diphenyl-2-picrylhydrazyl (DPPH) reduction by *P. major* hydroethanolic extract (PmHE). DPPH solution was incubated with different concentrations of *P. major* extracts and absorbance was measured at 517 nm (closed squares). Quercetin (0.1 mg/mL) was used as standard (closed circle). The results were expressed as percentage in relation to DPPH solution, considered as 100%. Inset: Electron paramagnetic resonance (EPR) spectra of DPPH radical obtained in the absence of the extract (control) or in the presence of 0.1 mg/mL PmHE.

DPPH (1,1-diphenyl-2-picrylhydrazyl) reduction assay is an efficient and rapid method to screen the scavenger activity of compounds or extracts *in vitro* [30]. Here, it was used to evaluate the antioxidant potential of PmHE at different concentrations (0.005 to 0.1 mg/mL). As observed in Figure 2, the increase in PmHE concentration resulted in bleaching of DPPH, reaching about 40% effectiveness at 0.1 mg/mL concentration. To exclude spectrophotometric artifacts and misinterpretation of this result, the electron paramagnetic resonance (EPR) technique was used. The inset in Figure 2 shows the decrease of DPPH signal intensity by PmHE, corroborating its free radical scavenger activity. Quercetin, which is a well-known free radical scavenger, was used as standard, and it was able to reduce more than 90% DPPH at 0.1 mg/mL concentration (Figure 2, closed circle). A concentration-response curve of DPPH reduction by quercetin has already been shown elsewhere [31]. Ethanolic solution presented a low yield of flavonoid extraction when compared with more hydrophobic solvents, such as dichloromethane and ethyl acetate [26], which probably resulted in this partial free radical scavenger activity exhibited by PmHE. It is noteworthy that DPPH reaction is predictive but not sufficient to input a therapeutic potential as antioxidant to an extract or compound, since the system does not resemble the physiological generation of free radicals [32]. In addition, a protective effect in the complex biological system (whole cells or *in vivo*) may be the sum of several chemical, physicochemical, and biological properties, and not only promoted by a free radical scavenger activity. Thus, we evaluated whether PmHE could protect isolated liver mitochondria against oxidative damage.

Mitochondria are the major source of ROS (reactive oxygen species) in respiring cells due to the continuous electron “escape” in the respiratory chain, and consequently these organelles have complex and efficient systems for ROS removal. Wherefore, the protective action of 0.1 mg/mL PmHE was evaluated in isolated mitochondria against the oxidative stress promoted by *t*-BOOH. At this concentration, PmHE alone (in the absence of oxidizing agent) did not alter any evaluated parameter in isolated mitochondria or cells (not shown).

**Figure 3:**
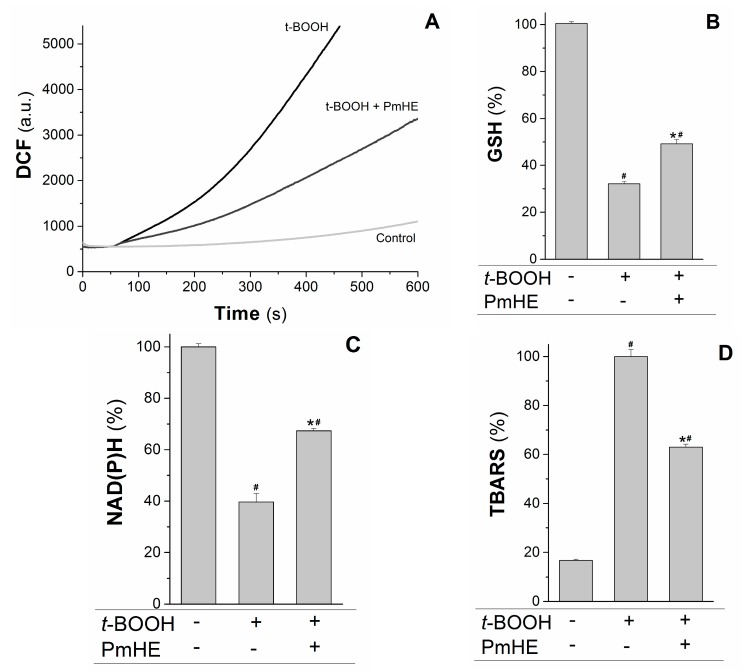
Protective effects of *P. major* hydroethanolic extract in isolated rat liver mitochondria exposed to the oxidant *t*-BOOH. Mitochondria (1 mg/mL) were incubated with PmHE (0.1 mg/mL) in the presence of 0.6 mM *t*-BOOH. (**A**) ROS (reactive oxygen species) generation was accompanied by monitoring DCF (2′,7′-dichlorodihydrofluorescein) fluorescence at 503/529 nm excitation/emission, respectively; traces are representative of (at least) three experiments with different mitochondrial preparations; (**B**) GSH (glutathione, reduced form) and (**C**) NAD(P)H levels measured fluorimetrically, as described in Materials and Methods. Data were presented as the mean of three experiments with different mitochondrial preparations; (**D**) Lipid peroxidation was as TBARS. Percentages were calculated by considering positive control as 100% and data were presented as mean ± S.E.M. of three experiments in triplicate with different mitochondrial preparations. * Statistically different from *t*-BOOH, and **^#^** statistically different from control (*p* < 0.05).

The kinetics of ROS production in succinate-energized isolated rat liver mitochondria was monitored using H_2_DCFDA (2′,7′-dichlorodihydrofluorescein diacetate), a non-fluorescent molecule that in the presence of ROS is rapidly oxidized to the fluorescent product DCF (2′,7′-dichlorodihydrofluorescein). As observed in Figure 3A, the addition of the oxidizing agent *t*-BOOH to the mitochondrial suspension increased the rate of ROS production, which was attenuated about 55% by the addition of PmHE. This decrease was accompanied by the significant protection of GSH (glutathione, reduced form) and NAD(P)H against *t*-BOOH-induced oxidation (Figure 3B,C, respectively).

GSH and NAD(P)H are pivotal substrates to enable the enzymatic elimination of peroxides through the glutathione peroxidase/reductase systems. Inside mitochondria, GSH is oxidized to the disulfide dimer form (GSSG) by glutathione peroxidase concomitantly with reduction of hydrogen peroxide to water and molecular oxygen. GSSG is recovered by glutathione reductase coupled to NAD(P)H oxidation [33,34]. Thus, the preservation of these substrates under oxidative conditions is relevant to maintain a reduced mitochondrial environment and, consequently, protect the organelle from oxidative damage. In order to assess the preservation of mitochondrial structures, the oxidative damage in mitochondrial membranes promoted by *t*-BOOH was evaluated in the presence and absence of PmHE. The extension of membrane lipid oxidation can be estimated by quantifying thiobarbituric acid reactive substances (TBARS) [35]. PmHE was able to prevent *t*-BOOH-induced lipid peroxidation about 40%, protecting mitochondrial membranes against oxidative damage (Figure 3D).

**Figure 4:**
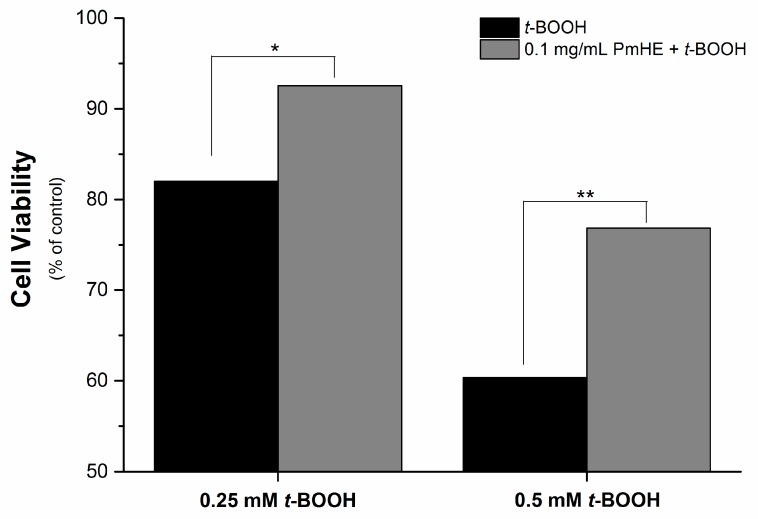
Cytoprotective effect of *P. major* hydroethanolic extract against *t*-BOOH-induced cell death. HepG2 cells (1.6 × 10^5^ cells/cm^2^) were incubated for 24 h in Dulbecco’s modified Eagle’s medium supplemented with 10% fetal bovine serum at 37 °C in a 5% CO_2_ atmosphere with 0.25 or 0.5 mM *t*-BOOH in the presence (gray bars) and absence (black bars) of 0.1 mg/mL PmHE. Both, *t*-BOOH and PmHE were added at the same time. */** Statistically different from 0.25 and 0.5 mM *t*-BOOH, respectively (*p* < 0.05).

It is well known that the imbalance of the mitochondrial redox state that results in disruption of calcium homeostasis and ATP depletion is associated with cell death [36]. Since PmHE presented a free radical scavenger activity, decreased ROS generation and prevented GSH, NAD(P)H, and lipid oxidation in isolated mitochondria, we challenged human liver HepG2 cultured cell with *t*-BOOH and estimated the cytotoxicity. As expected, *t*-BOOH decreased cell viability in a concentration-dependent manner (black bars) and the pre-incubation of cells with 0.1 mg/mL PmHE resulted in a significant protection against *t*-BOOH-induced cytotoxicity (Figure 4).

These results presented here on *P. major* hydroethanolic extract corroborate previous findings showing the scavenger activity of several Plantago species extracts with its antioxidant potential [19,20,37,38] and demonstrated that such activity is able to confer antioxidant protection in biological systems, by decreasing ROS production and maintaining the endogenous antioxidant status, and conferring cytoprotection in oxidative stress conditions.

## 3. Experimental Section

### 3.1. Plant Source and Extract Preparation

The dried aerial parts of *P. major* were acquired commercially from Panizza Laboratory (São Paulo, Brazil), accompanied of a certificate of analysis as a guarantee of origin. The extract was obtained with hydroethanolic solution (PmHE) using fractionated percolation described in the Brazilian Pharmacopoeia [39] and outlined in Mello *et al.* [26] using a 50% (*v*/*v*) ethanol solution (Merck Millipore Billerica, MA, USA, USA). An amount of 100 g of the dried plant was used to obtain 100 mL extract after the percolation process. After this, the ethanolic extract obtained was dried at 40 °C in the dark to evaporate the solvent and the crude extract was resuspended in dimethyl sulfoxide (DMSO, Sigma-Aldrich Co., St. Louis, MO, USA) at 25 mg/mL as a stock solution for the experiments. The final concentrations in the experiments contained 0.4% DMSO (or less) which presented no effect in isolated mitochondria or cells.

### 3.2. Total Phenolic Compounds and Flavonoid Quantification

Total soluble phenolics derivatives were determined according to the Folin-Ciocalteau method [40]. Samples (4 μL) were added to the reaction medium containing 0.05 mL of 7% (*v*/*v*) acetic acid, 0.05 mL of Folin-Ciocalteau’s reagent (Sigma-Aldrich), 87.5 μL of 20% (*w*/*v*) sodium carbonate and water to a final volume of 1 mL. After mixing, the reaction was incubated during 90 min at 30 °C in the dark and the absorbance was measured at 725 nm. Total phenolic content was determined based on a standard curve with gallic acid (Sigma-Aldrich). To quantification of flavonoids, an aliquot of the samples were incubated in a medium containing 60 μL of glacial acetic acid, 1 mL of 12% pyridine:H_2_O:AlCl_3_ (17:80:3) solution, 1.24 mL of 50% DMSO for 5 min at 25 °C. The reaction product was determined spectrophotometrically at 420 nm and the flavonoid concentration was estimated based on a standard curve with quercetin (Sigma-Aldrich).

### 3.3. HPLC Analysis

The separation and qualitative identification of gallic acid and the flavonoids luteolin (LUT), catechin (CAT), taxifolin (TAX), and galangin (GAL) in *P. major* extract was carried out in a Agilent 1200 HPLC (Agilent Technologies, Inc., Santa Clara, CA, USA). An Inertsil ODS-3 C18 column of 250 × 4.6 mm length and a 5.0-mm particle size was used and detection was carried out at 245, 254, 280, 335, and 410 nm using a MWD spectrophotometric detector. The elution was performed using a mobile phase consisting of 0.1% aqueous acetic acid (A), acetonitrile (B) and tetrahydrofuran (C), and the gradient conditions were: 0–30 min, 97% A:3% B–75% A:25% B; 30–50 min, 62% A:38% B; 50–70 min, 40% A:60% B; 70–85 min, 30% A:70% B; 85–105 min, 20% A:65% B:15% C, 105–120 min, 97% A:3% B. The injection volume of extract was 5 µL (diluted in DMSO, 10 mg/mL) and the flow rate was 0.6 mL/min at 25 °C. The peaks were identified by comparison of the retention time of standard isolated compounds (Sigma-Aldrich) by analytical HPLC under the same experimental conditions.

### 3.4. DPPH Reduction Assay

Reduction of the 1,1-diphenyl-2-picrylhydrazyl radical (DPPH) by *P. major* extract was determined spectrophotometrically at 517 nm [41]. Different concentrations of extract were incubated for 5 min at 30 °C in 1.5 mL of 40 mM sodium acetate pH 5.5 followed by the addition of 1 mL absolute ethanol containing DPPH (0.1 mM final concentration). Quercetin, a well know free radical scavenger, was used as standard. The reduction of DPPH was confirmed by electron paramagnetic resonance [42]. The extract (0.1 mg/mL) was incubated in 40 mm sodium acetate buffer (pH 5.5), followed by the addition of a methanolic DPPH solution (0.1 mM final concentration). The EPR spectra were recorded on a Bruker X-band spectrophotometer. The measurements were repeated three times for each sample. EPR measurement conditions included: 2 × 10^3^ receiver gain, 0.1 mT modulation amplitude, 10.0 mT sweep width, 349.5 mT centre field, 20.48 ms time constant, 80 ms conversion time, 9.7968 GHz microwave frequency, 100 KHz modulation frequency, and 10 mW microwave power. After acquisition, the spectra were simulated by BrukerWin-EPR software, version 2.11, and had their signals double integrated to obtain relative concentrations. The intensities are expressed in arbitrary units.

### 3.5. Isolation of Rat Liver Mitochondria

Liver mitochondria were isolated by conventional differential centrifugation from adult male Wistar rats as described previously [43]. All experiments involving animals were previously approved by the Ethical Committee of Animal Experimentation of University of Mogi das Cruzes (CEUA).

### 3.6. Reactive Oxygen Species (ROS) Generation

Mitochondrial ROS production was estimated by measuring the changes in the 2′,7′-dichlorodihydrofluorescein diacetate fluorescence [44]. Mitochondria (1 mg/mL) were incubated in a medium 125 mM sucrose, 65 mM KCl, 10 mM HEPES-KOH, pH 7.4, plus 1 µM H_2_DCFDA at 30 °C in the presence or absence of the extract of the 0.1 mg/mL PmHE. After addition of 5 mM potassium succinate (plus 2 μM rotenone), DCF fluorescence was accompanied at 503/529 nm excitation/emission wavelength pair in a Fluorescence Spectrophotometer Hitachi F-2500 (Hitachi, Tokyo, Japan). An amount of 0.6 mM *t*-BOOH plus 10 μM CaCl_2_ was used to induce the oxidative stress.

### 3.7. Mitochondrial GSH and NAD(P)H Levels

After a 30 min incubation at 30 °C in the presence or absence of the extract of the 0.1 mg/mL PmHE in a medium containing 125 mM sucrose, 65 mM KCl, 10 mM HEPES-KOH, pH 7.4, plus 5 mM potassium succinate (plus 2 μM rotenone) and 0.6 mM *t*-BOOH plus 10 μM CaCl_2_, mitochondrial suspension (1 mg/mL) was treated with 0.5 mL of 13% trichloroacetic acid and centrifuged at 900× *g* for 3 min. Aliquots (100 µL) of the supernatant were mixed with 2 mL of 100 mM sodium phosphate buffer pH 8.0 containing 5 mM EGTA followed by the addition of 0.1 mL of 1 mg/mL *o*-phthalaldehyde (OPT). End-point fluorescence was measured 15 min later using the 350/420 nm excitation/emission wavelength pair with excitation/emission slit of 5 nm [45]. The intrinsic fluorescence of NADH/NADPH was accompanied at 366/450 nm excitation/emission wavelength pair in mitochondrial suspension (1 mg/mL) incubated in a medium containing 125 mM sucrose, 65 mM KCl, 10 mM HEPES-KOH, pH 7.4, plus 5 mM potassium succinate (plus 2 μM rotenone) and 0.6 mM *t*-BOOH plus 10 μM CaCl_2_.

### 3.8. Lipid Peroxidation

The lipid oxidation of mitochondrial membranes was estimated by measurement of thiobarbituric acid reactive substances generation. Mitochondria (1 mg/mL) were incubated in a medium containing 130 mM KCl, 10 mM HEPES-KOH pH 7.4 plus 5 mM potassium succinate (+2 μM rotenone) for 30 min at 37 °C in the presence or absence of the extract of the 0.1 mg/mL PmHE. To initiate the lipid oxidation chain reaction an amount of 0.6 mM *t*-BOOH plus 10 μM CaCl_2_ was used. For TBARS determination, 1 mL of 1% TBA, 0.1 mL of 10 M NaOH and 0.5 mL of 20% H_3_PO_4_ were added to the mixture reaction followed by further incubation for 20 min at 85 °C. TBARS were extracted with 2.0 mL of *n*-butanol and the absorbance was read at 532 nm.

### 3.9. Cell Culture

Human liver HepG2 cells were cultivated in high-glucose Dulbecco’s modified Eagle’s medium (DMEM; Sigma Chemical Co. St. Louis, MO, USA) supplemented with 10% fetal bovine serum (heat inactivated, South American origin; GIBCO-Invitrogen Corp., Grand Island, NY, USA), 100 U/mL penicillin (GIBCO-Invitrogen Corp. Gaithersburg, MD, USA), and 100 μg/mL streptomycin (GIBCO-Invitrogen Corp.) at 37 °C in a 5% CO_2_ atmosphere (Sanyo MCO-20AIC incubator; Sanyo Electric Co., Ltd., Osaka, Japan). For the experiments, cells were washed twice with a calcium- and magnesium-free buffered saline solution (CMF-BSS), detached from the flasks with trypsin/EDTA (GIBCO-Invitrogen Corp.), and resuspended in the supplemented media.

### 3.10. Cytotoxicity Assay

The *t*-BOOH-induced cytotoxicity was evaluated by the MTT reduction test in cultured liver cells. HepG2 cells (1.6 × 10^5^ cells/cm^2^) were added to 96-well microplates (0.2 mL final volume) and incubated with 0.25 or 0.5 mM *t*-BOOH in the presence or absence of 0.1 mg/mL PmHE for 24 h at 37 °C and 5% CO_2_ atmosphere. After incubation time, 0.25 mg/mL MTT (3-[4,5-dimethylthiazol-2-yl]-2,5-diphenyl tetrazolium bromide) was added followed by 4 h incubation. Then 0.1 mL of 10% SDS (sodium dodecyl sulfate), prepared in 0.01 M HCl, was added and incubated overnight to dissolve the formazan crystals. Plates were read at 570 nm (Microplate Reader Biotek ELX 800, BioTek Instruments, Inc., Vermont, VT, USA) and the cell viability was determined in relation to control (absence of *t*-BOOH and PmHE) considered as 100%.

### 3.11. Data Analyses

All experiments were performed at least three times (three different mitochondrial preparations), each one in triplicate. Results were analyzed using one-way analysis of variance (ANOVA) followed by Dunn’s *post-hoc* test for multiple comparisons using GraphPad Prism^®^ 3.0 (GraphPad Software, Inc., California, CA, USA). A significance level of *p* < 0.05 was considered as significantly different. The graphics were constructed using Microcal (TM) Origin^®^ version 6.0 (Microcal Software Inc., Northampton, MA, USA).

## 4. Conclusions

On consideration that ethanol-water solutions represent the common method used in folk medicine to obtain plant extracts, the efficiency of such a system was shown here for the extraction of active antioxidant compounds of *P. major*. Such antioxidant activity exhibited by *P. major* hydroethanolic extract was able to protect isolated mitochondria against *t*-BOOH-induced oxidative damage. The cytoprotective effect of PmHE against *t*-BOOH induced cell death was also shown. These findings contribute to the understanding of the health benefits attributed to *P. major*.
